# Case Report: Tracheal infiltration with wheezing revealing Hodgkin's disease

**DOI:** 10.12688/f1000research.130928.1

**Published:** 2023-04-14

**Authors:** Selsabil Daboussi, Asma Saidane, Samira Mhamdi, Nouha boubaker, Chaabane Mariem, Chiraz Aichaouia, Ghedira Hela, Fehmi Msadek, Moetemri Zied

**Affiliations:** 1 University of Tunis El Manar, Tunis, Tunisia; 2 Pneumology Department, Military Hospital of Tunis, Tunisia, 1008, Tunisia; 3 Hematology Department, Military hospital of Tunis,, Tunis, 10008, Tunisia

**Keywords:** Hodgkin’s disease, wheezing, trachea, chemotherapy

## Abstract

Hodgkin's disease with an initial tracheobronchial involvement is extremely rare. The symptoms might be misleading, resulting in a diagnosis delay. We report the case of a 38-year-old woman with a one-month history of wheezing and a non-productive cough. The physical examination revealed a good general state of health, bilateral diffuse wheezing and supra-clavicular lymphadenopathy. The adenopathy biopsy's histopathology revealed Hodgkin's disease. The whole body FDG-PET scan was an important tool to assess the diagnosis as well as for the staging. The patient was treated with chemotherapy. Another unusual aspect is the tracheobronchial metastasis confirmed by a bronchial biopsy. Thus, our patient was put on a second-line chemotherapy. She died one year after the initial diagnosis. To conclude, it is a rare case of an Hodgkin lymphoma with a tracheobronchial relapse. It should be considered in the differential diagnosis of a tracheal tumor.

## Background

Hodgkin's disease cases with tracheobronchial involvement are rare. ^1^ The symptoms are not specific and may mimic many other diseases like asthma or chronic obstructive pulmonary disease (COPD), which delays the lymphoma diagnosis as well as the treatment. ^1^^,^^2^ It is now recognised as a treatable neoplasia. Thus, it is worth mentioning this entity for clinicians especially after the recent therapeutic progress We report the case of a 38-year-old woman with a one-month history of wheezing and a non-productive cough unresponsive to bronchodilators revealing Hodgkin's disease.

## Case presentation

A 38-year-old female presented to our department with a one-month history of dry cough and wheezing. She has never smoked. She has not any comorbidities or allergies. She is a stay-at-home spouse and is white. She has visited the emergency room several times in the last month for chest wheezing. She received beta2-mimetics nebulizations, without any improvement. The physical examination at admission revealed a good general state. Respiratory auscultation revealed bilateral diffuse wheezing. Oxygen saturation was (98 %) on room air. The examination of the ganglionar areas detected peripheral bilateral fixed supra-clavicular lymphadenopathy measuring about (2cm of diameter). There was no hepatosplenomegaly. The neurological examination was normal.

**Figure 1.**
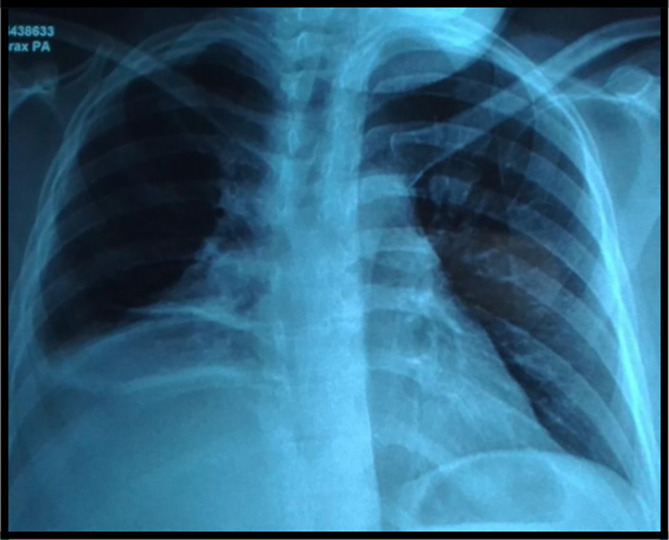
Chest X-ray showing a retractile opacity in the right chest with hilar enlargement.

The chest x-ray showed a retractile opacity in the right chest with a hilar enlargement ( Figure 1).

**Figure 2.**
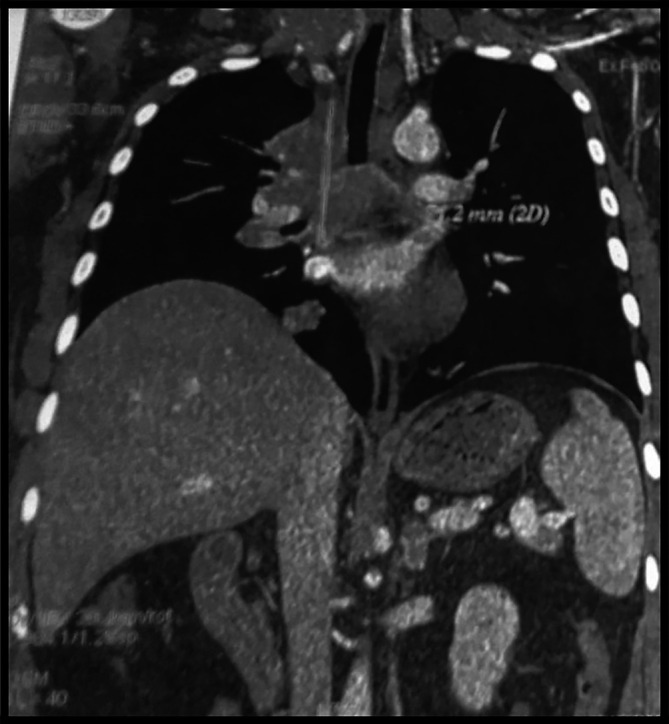
Coronal section of the computed tomography chest demonstrating a bulky tumor invading the carina, the right main bronchus, the pericardium with a subpleural right node.

**Figure 3.**
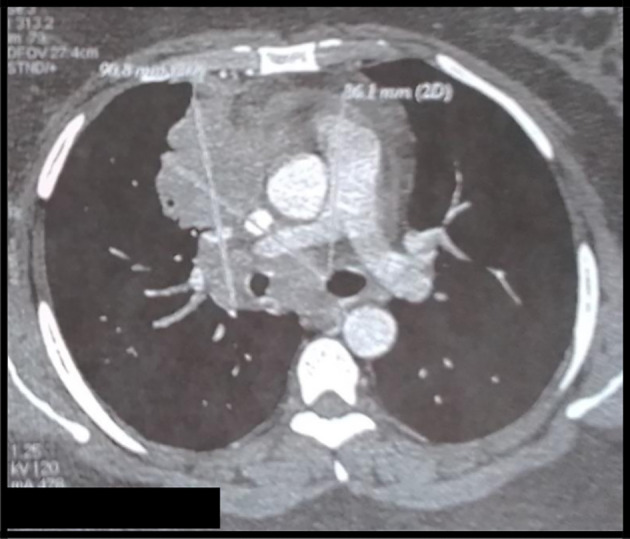
CT mediastinal section showing an important mediastino-hilar mass infiltrating the right main bronchus.

The body computed tomography (CT) scans revealed a bulky mediastino-hilar tumor in the right chest, measuring (90 x 85 x 71 mm), invading the right main bronchus, extending to the vena cava and infiltrating the pericardium with a subpleural right node ( Figures 2 and 3). Bronchoscopy showed a polypoid mass located in the carina, involving the right main bronchus. Cytology revealed malignant cells. Histopathology of the bronchial biopsy did not show any malignant lesions. In addition, we performed a biopsy of the supra-clavicular adenopathy. Histopathology revealed an intense staining for CD15 and CD30 with large multinuclear reed Sternberg cells. Therefore, the diagnosis of an Hodgkin lymphoma was confirmed.

**Figure 4.**
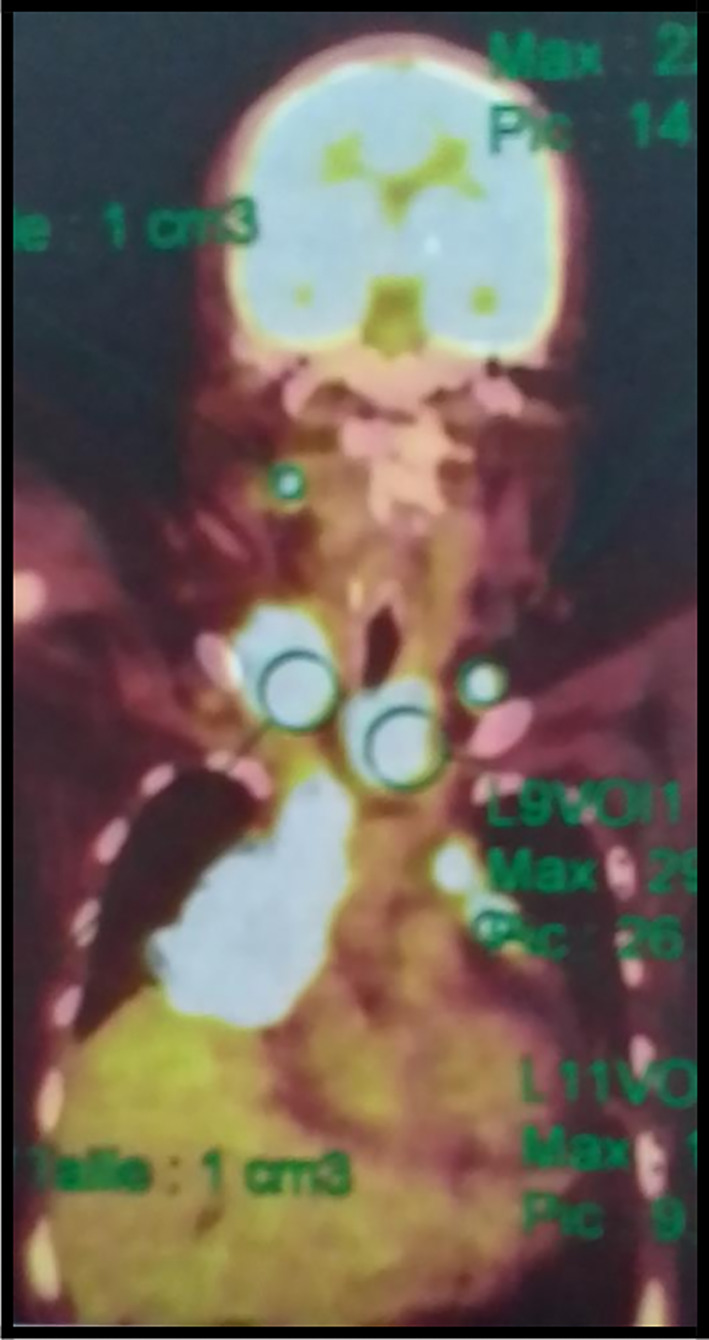
A PET scan image showing hypermetabolic lymph nodes.

**Figure 5.**
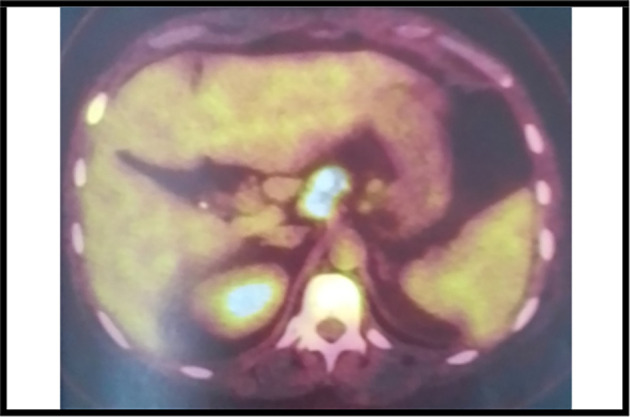
A PET scan image showing the subphrenicganglionar fixation of the FDG.

The 18-fluorodeoxyglucose-positron emission tomography (FDG-PET) showed many sites of activity of the disease, with a high metabolic fixation of the FDG in the lymph node stations (1L,3,5,6,7,8,9,10,11R) as well as subphrenic adenopathy. Besides, it revealed an intense endotracheal metabolic fixation ( Figures 4, 5). Moreover, the bone marrow biopsy was negative.

The patient received 4 courses of an escalated BEACOPP chemotherapy protocol (Bleomycin, Etoposide, Adriamycin, Cyclophosphamide, Vincristine, Procarbazine, Prednisone) as detailed below ( Table 1).

**Table 1.** The escalated BEACOPP protocol (21 days).

Day	Drug	Dose	Route
Day 1	Adriamycin	35 mg/m ^2^	IV bolus
Day 1	Mesna	1000 mg/m ^2^ (prior to cyclophosphamide)	IV infusion
Day 1	Cyclophosphamide	1250 mg/m ^2^	IV infusion
Day 1	Mesna	1000 mg/m ^2^ (4 hours after cyclophosphamide)	IV infusion
Day 1 up to Day 3	Etoposide	200 mg/m ^2^	IV infusion
Day 1 up to Day 7	Procarbazine	100 mg/m ^2^	PO
Day 1 up to Day 7	Prednisolone	40 mg/m ^2^	PO
Day 8	Vincristine	1.4 mg/m ^2^	IV infusion
Day 8	Bleomycin	10 mg/m ^2^	IV infusion
Day 9 up to Day 13	G-CSF	5 μg/kg	SC

She was readmitted in our department 2 months after for acute respiratory failure with wheezing. Bronchoscopy revealed a bulky stenosing polypoid mass, measuring about 3 cm of diameter, rising from the carina extending to the trachea and to the main bronchus that remains partially patent. Histopathology revealed large cells associated with an inflammatory granuloma of (lymphocytes, eosinophils, neutrophils) and a positive staining for CD 15 and CD 30. Therefore, we concluded to a tumoral progression with an endotracheobronchial relapse.

The decision of the multidisciplinary medical team was to put the patient under a second-line chemotherapy: IGEV Regimen as a salvage therapy (ifosfamide, gemcitabine, vinorelbine and methylprednisolone). Moreover, injected Chest CT scans revealed a segmental pulmonary embolism. So, she received a curative anticoagulant treatment. She died one year after the initial diagnosis.

## Discussion

It is well known that Hodgkin lymphoma usually involves the mediastinum. However, pulmonary involvement is less common and can be observed in the lymph nodes, parenchyma, and tracheobronchial tree. ^1^

It is worth mentioning that Hodgkin lymphoma with a tracheobronchial involvement remains extremely rare. ^1^ Patients usually present with respiratory symptoms (such as dyspnea, wheezing and cough), fever, and weight loss. ^2^ The diagnosis may be challenging because the symptoms are not specific. About (30%) of the patients have more than 6-months diagnosis delay. ^3^ We should consider a tracheobronchial tumor in the differential diagnosis when the patient presents to our department with asthma symptoms without any improvement after bronchodilators. In our case, the patient complained of a dry cough and chest wheezing mimicking an asthma attack. She received Beta2-mimetics without any improvement.

Atelectasis is the most frequent radiological finding, occurring in (2/3) of patients. Besides, the chest CT scan may reveal a solitary hilar mass or an obstructive emphysema. Mediastinal or hilar lymphadenopathy are often present. ^4^

Endoscopy may show ulceration, mucosal infiltrate, or a polypoid mass. ^1^ Our patient had a polypoid mass rising from the trachea extended to the main bronchus.

Histopathology shows typically large Sternberg cells with positive staining for CD15 and CD30. ^3^ The diagnosis was made thanks to a biopsy of the supra-clavicular adenopathy in this case. Recent studies suggest that endobronchial ultrasound (EBUS) may be useful in the diagnosis and staging of Hodgkin lymphoma with endobronchial involvement to avoid mediastinoscopy. ^5^

It is worth mentioning that the PET scan is a reliable imaging tool for the diagnosis as well as for the staging of lymphoma especially in this case because mediastinal adenopathy may be due to infectious or inflammatory diseases. ^6^^,^^7^ In our case, this exam showed an intense metabolic fixation FDG in many ganglionar areas as well as an endotracheobronchial fixation. It is also very interesting for the follow-up.

Treatment is commonly based on chemotherapy with or without radiotherapy. It depends strongly on the extent of the disease, the general state and the comorbidities. ^8^ Complete resection of the stenotic tracheal tumor may be performed in patients with critical airway obstruction thanks to interventional procedures such as rigid bronchoscopy with a stent placement, photodynamic laser therapy, laser therapy with a neodymium: yttrium-aluminum-garnet laser (Nd-YAG) and photodynamic laser therapy. ^9^^,^^10^

Relapsed Hodgkin lymphoma is another challenging problem for clinicians especially when it affects the tracheobronchial tree as seen in our case. Prognostic Factors associated to the relapse of the disease have been recently identified including: a poor performance status (PS), an age greater than 50 years old, failure to achieve remission after an initial therapy, anemia and an advanced lymphoma with a clinical staging (III or IV). ^11^

## Conclusion

To conclude, we report a very rare case of lymphoma with an endobronchial involvement. We do underline the importance of considering Hodgkin lymphoma in the differential diagnosis of asthma symptoms without any improvement after bronchodilators or in the case of a tracheal tumor. Further studies are required in order to highlight the prognostic factors in order to improve the outcomes.

## Consent

Written informed consent for publication of their clinical details and/or clinical images was obtained from the family of the patient.
