# Supplementary material for: Case Report: Tracheal infiltration with wheezing revealing Hodgkin's disease
Source: F1000Res. 2023 Sep 11;12:404. Originally published 2023 Apr 14. [Version 2] doi: 10.12688/f1000research.130928.2 (PMC10521064; doi:10.12688/f1000research.130928.2)
Supplement: Peer review history

# Reviewer report: Reviewer response for version 2

**DOI:** 10.5256/f1000research.155070.r205342

**Published:** 2023-09-15

**Authors:** Jeffrey W Craig

**Affiliations:** 1 University of Virginia Health System, Charlottesville, Virginia, USA

I appreciate the attention given by Daboussi *et al. *to the previous reviewer comments. Although not all suggestions for improvement were acted upon, it does appear that the authors made sincere efforts to revise this manuscript in a positive direction.

Are enough details provided of any physical examination and diagnostic tests, treatment given and outcomes?

Yes

Is the case presented with sufficient detail to be useful for other practitioners?

Partly

Is sufficient discussion included of the importance of the findings and their relevance to future understanding of disease processes, diagnosis or treatment?

Partly

Is the background of the case’s history and progression described in sufficient detail?

Partly

Reviewer Expertise:

Diagnostic hematopathology; lymphoma biology

I confirm that I have read this submission and believe that I have an appropriate level of expertise to confirm that it is of an acceptable scientific standard.

# Reviewer report: Reviewer response for version 1

**DOI:** 10.5256/f1000research.143726.r190574

**Published:** 2023-08-16

**Authors:** Jeffrey W Craig

**Affiliations:** 1 University of Virginia Health System, Charlottesville, Virginia, USA

This manuscript reports a single case of classic Hodgkin lymphoma in a young adult female presenting with one month of dry cough, wheezing and palpable supraclavicular lymphadenopathy. Initial imaging revealed extensive mediastinal disease (up to 90 mm) with infiltration of the pericardium and right mainstem bronchus, the latter of which was also visualized bronchoscopically as a polypoid carinal mass. Diagnosis was based on histopathology of an involved lymph node. PET scan showed extensive foci of hypermetabolism, including the tracheal lesion and lymph nodes above and below the diaphragm. The patient was treated with front-line BEACOPP therapy; however, primary refractory disease was confirmed just two months later. A salvage IGEV regimen was then employed, but the patient expired roughly one year after the initial diagnosis.

 I have the following general comments/criticisms of the manuscript.

1. I think it’s very misleading to describe or compare this case to primary endobronchial Hodgkin lymphoma, as this patient presented with palpable lymphadenopathy and more widespread disease on imaging. Lung/airway involvement by Hodgkin lymphoma is not particularly uncommon, especially in young adult patients with bulky intrathoracic disease. The clinical presentation did include airway symptoms, but the diagnosis was readily obtained via conventional lymph node biopsy.
2. The current WHO & ICC nomenclature for this entity is classic Hodgkin lymphoma, not Hodgkin’s disease (antiquated terminology). I strongly recommend the addition of a hematopathologist to the authorship list, with the goal of improving the descriptions of the histopathologic findings. Further, the manuscript would likely benefit from having a microscopic image in support of this diagnosis, as well as formal staging information (presumably stage IIIA/IVA).
3. The manuscript could use another round of proofreading for language, grammar and punctuation.

Are enough details provided of any physical examination and diagnostic tests, treatment given and outcomes?

Yes

Is the case presented with sufficient detail to be useful for other practitioners?

Partly

Is sufficient discussion included of the importance of the findings and their relevance to future understanding of disease processes, diagnosis or treatment?

Partly

Is the background of the case’s history and progression described in sufficient detail?

Partly

Reviewer Expertise:

Diagnostic hematopathology; lymphoma biology

I confirm that I have read this submission and believe that I have an appropriate level of expertise to state that I do not consider it to be of an acceptable scientific standard, for reasons outlined above.

# Response

**Published:** 2023-08-23

**Authors:** DABOUSSI SELSABIL

**Affiliations:** Tunis El Manar University, Tunisia

**Dear Dr. Craig,**

           Thanks a lot for your interest! We have included two specialists in clinical Hematology and a specialist in anatomopathology in order to improve the description of the lesions. Moreover, our therapeutic protocol was made according to the recent guidelines.

 Extranodal lymphoma is not rare itsself. However, a primary clinical presentation of an extranodal lymphoma with an associated tracheobronchial involvement is so rare in antemortem series. It is often diagnosed in patients followed up for lymphoma during the follow up or in autopsy series. In fact,the diagnosis is so challenging given that the symptoms may mimic a wide spectrum of diseases like asthma or COPD as shown in our case report. Indeed, the bronchial biopsy is often negative. Thus, we aimed to highlight the importance of interventional endoscopy procedures in the initial diagnosis and during  the follow up. Moreover, the tracheobronchial relapse is unusual. As you know, the Hodgkin lymphoma invades typically the lymph nodes.

         Finally, we aimed to highlight the atypical manifestations of lymphoma in order to increase the awareness of clinicians and improve the prognosis. Since an early diagnosis implies an early treament, it would alleviate the burden of the disease. Besides, we really hope to share this case with other experts in order to find out better therapeutic stratgies. Our patient died despite a combined chemotherapy and a strict follow-up. The treatment of an advanced stage lymphoma is usually aggrssive with a poor prognosis. The median survival does not exceed some months in our developing countries. It would encourage experts to carry on new researchers in this field.

 Kind regards

 Saidane Asma

 Military hospital

# Reviewer report: Reviewer response for version 1

**DOI:** 10.5256/f1000research.143726.r171452

**Published:** 2023-05-10

**Authors:** Ruwaida Ben Musa

**Affiliations:** 1 University of Missouri, Columbia, MO, USA

It’s an interesting case report that addressed the importance of considering a tracheal tumor in the differential diagnosis of bronchial asthma when the patient presents with asthma symptoms without any improvement after bronchodilators. Although Hodgkin lymphoma with a tracheobronchial involvement remains extremely rare, the clinician should be aware of that and consider it in the differential diagnosis in order to diagnose the tumor in the early stage of the disease and save the patient's life.   

 I have a number of comments that the authors may find useful:

1. The background of the case’s history was very short, I encourage the authors to give more details about Hodgkin's disease. As well as more details about the patient's history in the (case presentation) would be helpful such as nationality, if she is married, occupation/environment, history of passive smoking, alcohol, family history, and other risk factors. Also, if the patient presented with any other symptoms such as unexplained fever or weight loss, fatigue, night sweating, and chest pain?  
2. The authors state that the patient visited the emergency room several times, I am wondering if the ER doctor performed a complete physical examination including the lymph node examination in order to discover the supra-clavicular lymphadenopathy. I am also wondering if the patient does not improve by the Beta agonist treatment, why the ER doctor does not order the chest X-ray during that time to exclude any other diseases?  
3. During respiratory auscultation usually, we used the term bilateral diffuse rhonchi instead of wheezing. Also, I suggest the authors include more details on the physical examination, such as the vital signs during the time of admission, and the general physical examination if the patient was conscious, oriented, dyspneic, tachypneic, anemic, any cyanosis and lower limb edema?
4. On page #3, the authors mentioned (PET) scan that showed many sites of activity of the disease, with a high metabolic fixation of the FDG in the lymph node stations (1L,3,5,6,7,8,9,10,11R). Are the authors using the International Association for the Study of Lung Cancer (IASLC) to define the stations? Could you explain it in the paper if this is the case? Also, the patient was at which stage when Hodgkin's lymphoma was diagnosed?  
5. For Fig #3; to be more clear, I think it's better to use the arrow to show the site of the bulky tumor which invades the carina. The same thing with Fig #4, the CT mediastinal section, please add more description in the legend that would be helpful.  
6. For Fig #4, looks like a blurry image and is not clear to me with little description in the legend. I encourage the authors to mention more details in each figure and use arrows and landmarks to be easier to understand the regions that are involved with tumors.  
7. I am wondering as I did not see any blood test results or requested such CBC, ESR, CRP, Blood film, etc.? Have physicians requested? I think it’s worth mentioning it in the case report by making a table of the blood test results even if it is normal.  
8. I think more details on the BEACOPP chemotherapy protocols would be helpful such as regimens, dosage, and for how long the patient received it. Additionally, if there was any follow-up CT or PET scan done after the patient received chemotherapy. Does the patient receive any radiotherapy or immunotherapy? 
9. It appears the patient does not have regular follow-ups with her doctor after receiving the chemotherapy as she developed acute respiratory failure as a result of bulky stenosing polypoid mass which was diagnosed by bronchoscopy and unfortunately she also developed segmental pulmonary embolism. I encourage the authors to address if the patients were on regular follow-ups in the case presentation section or not. 

Are enough details provided of any physical examination and diagnostic tests, treatment given and outcomes?

Partly

Is the case presented with sufficient detail to be useful for other practitioners?

Yes

Is sufficient discussion included of the importance of the findings and their relevance to future understanding of disease processes, diagnosis or treatment?

Yes

Is the background of the case’s history and progression described in sufficient detail?

Partly

Reviewer Expertise:

My area of expertise in research is the neuronal control of the Cardiorespiratory system during exposure to hypoxia by using the transgenic animals model.

I confirm that I have read this submission and believe that I have an appropriate level of expertise to confirm that it is of an acceptable scientific standard.
